# Nurses’ and patients’ experiences of family planning services in a rural district, South Africa

**DOI:** 10.4102/phcfm.v15i1.3732

**Published:** 2023-05-10

**Authors:** Kartik Naidoo, Louis S. Jenkins

**Affiliations:** 1 Department of Family and Emergency Medicine, Faculty of Medicine and Health Sciences, Stellenbosch University, Cape Town, South Africa; 2 Primary Health Care Directorate, Department of Family, Community and Emergency Care, Faculty of Health Sciences, University of Cape Town, Cape Town, South Africa; 3 Department of Family and Emergency Medicine, George Provincial Hospital, Western Cape Department of Health, George, South Africa

**Keywords:** family planning, contraception, primary health care, clients, nurses, rural, experiences

## Abstract

**Background:**

Family planning (FP) is a key component of primary health care (PHC). Nurses are the first source of FP information to women outside their social context. There is a paucity of research regarding clients’ lived experiences of FP, particularly understanding both the client’s and the healthcare worker’s experiences in the same clinical context and community.

**Aim:**

This study aims to explore the lived experiences of nurses and female clients regarding FP services at PHC clinics.

**Setting:**

Two PHC clinics in a rural sub-district in South Africa.

**Methods:**

A descriptive qualitative study using semi-structured interviews was conducted. Clients and nurses were selected using criterion-based purposive sampling and interviewed by female research assistants in a home language in a private setting. Transcription and translation of audio recordings were done. Data were analysed inductively using the framework method.

**Results:**

Ten clients and eight nurses were interviewed, with an equal number from each clinic. The median age of clients was 28.5 years and of nurses was 47.5 years. Four themes emerged: (1) Stigma, culture and the teenage girl; (2) Bad effects – the Big Five, clustered around weight changes, blood blockages and abnormal bleeding, pain, fertility and cancer; (3) FP social dynamics; and (4) FP and the health system.

**Conclusion:**

Family planning is highly moralised and stigmatised. Negative effects of FP were not adequately recognised by the health system. Family planning outreach into the community and dedicated FP resources at clinics were suggestions to improve the service.

**Contribution:**

This work helps to better understand patients’ experiences of family planning services.

## Introduction

Family planning (FP) forms a key component of primary health care (PHC) and contributes significantly to reducing maternal and child mortality.^1,2,3^ This is reinforced by the United Nation’s sustainable development goals, with member states committing to provide universal access to sexual and reproductive healthcare, including FP.^4^ This includes monitoring the proportion of women having their FP needs met.^5^

A South African (SA) survey in 2016 showed that the overall use of modern contraceptives (female and male sterilisation, intrauterine contraceptive devices [IUDs], implants, injectables, pills, male and female condoms, and emergency contraception) by women aged 15–49 years was 58%, but 18% of women still had unmet contraception needs.^6^ Injectables available in the SA public health sector are depo-medroxyprogesterone acetate given 3-monthly and norethisterone enanthate given 2-monthly.^7^ The IUD mainly refers to the copper-releasing IUD which is what is available in the public health sector.^7^ Implants refers to the Implanon NXT (Nexplanon), which is a subdermal implant containing etonogestrel.^7^ Of the 58% of women using contraceptives, 15% relied on male condoms, 25% used injectables, while only 5% used long-acting reversible contraceptives (LARCs) such as implants and IUDs.^6^ This illustrates that women do not always choose the most effective form of contraception, particularly LARCs.^8^

The couple-year protection rate (CYPR) measures the proportion of women aged 15–49 years who are protected against unplanned pregnancies for a year using modern contraception, including sterilisation.^9^ Over the past 5 years, local data showed that 56% of the CYPR was contributed by condoms alone, while injectables contributed 18% and LARCs contributed only 9%.^10^

Studies aiming to improve the uptake of LARCs have identified the need to understand women’s perspectives on a particular method. Interventions that improved knowledge of LARCs did not necessarily translate into improved uptake.^11^ A deeper understanding of women’s attitudes and beliefs regarding FP is needed rather than just knowledge.^12^ This is exemplified by the fact that barriers to FP include: (1) lack of information, (2) fear of side effects, (3) unacceptability of amenorrhoea, (4) beliefs regarding different methods, (5) experiences of other women, (6) delayed return to fertility, (7) male partner approval and (8) fear of judgement.^12,13,14,15^

When engaging with FP services at a (PHC) clinic, a female client first encounters a nurse, the majority of whom are female and are likely from the same community. This nurse becomes the first source of FP information to the client outside of her social context. She may have herself used FP and may also bring to this consultation her personal experiences, expectations and biases.^15,16^ Younger clients often feel judged by older nurses and feel that there is not enough time to have their FP questions answered.^14^ Nurses, correspondingly, feel pressured by the number of clients, and there is a tension between the quality of service they would like to provide and what they are able to.^17^ The uptake of LARCs is also reduced by health service issues, such as client-staff ratios and the capability of healthcare workers.^11^ Healthcare workers may not promote LARCs, in particular IUDs, because of lack of knowledge, lack of skills to insert them, perceived risks of infections and assumptions about the clients’ perceptions of the device.^18^ Efforts to improve client-centredness and the quality of information provided through structured counselling tools may improve the uptake of LARCs and FP in general, but tend to demand more time from healthcare workers.^19^ Therefore, programmes aiming to improve service provision need to take into account the actual experiences and concerns of healthcare workers in providing FP services.^11,12^

For a service that is so dependent on personal decision-making, there is a paucity of research seeking to understand women’s lived experiences of FP.^14^ Surveys may fail to identify rich and relevant data regarding perceptions and expectations.^18^ There is evidence that shows that women’s negative attitude towards FP may be an important barrier preventing the uptake of FP services, highlighting the need to understand this more deeply.^20^ Studies have typically focused on younger women, human immunodeficiency virus (HIV)-positive women, postpartum contraception and specific FP methods.^15,17,21,22^ There is a risk to miss more nuanced reasons why women in general would choose against FP. There are even fewer studies that attempt to explore both the client’s experiences and the healthcare worker’s experiences. Understanding the experiences of both clients and healthcare workers in a single clinical or social context is important, as a healthcare intervention such as FP occurs within a relationship between the patient and the healthcare worker, and both influence the outcome.^15,17^ Finally, in terms of the social value of the study, there are no published studies that explore the FP experiences of both clients and nurses in a rural district in the Western Cape of South Africa. The aim of this study was to explore the lived experiences of both nurses and female clients regarding FP services at public sector PHC clinics in a rural sub-district in South Africa.

## Research methods and design

### Study design and setting

A descriptive qualitative study using individual semi-structured interviews was conducted at Alma and D’Almeida clinics, two of the largest PHC clinics in Mossel Bay, a rural sub-district in the Garden Route district of the Western Cape, South Africa. Mossel Bay had an estimated population of 95 225 people in 2019, with 53% being female.^23^

D’Almeida clinic, attended by 4000–5000 patients monthly, had three clinical nurse practitioners (CNPs), two of whom could administer all FP methods. There were three professional nurses (PNs), three enrolled nurses (ENs) and two enrolled nursing assistants (ENAs). The ENAs could also administer FP methods under supervision. Alma clinic, attended by 7000–8000 patients monthly, had five CNPs, all of whom could administer all FP methods. There were four PNs, five ENs and one ENA. At both clinics, the PNs and ENs could provide follow-up FP methods once initiated either by a CNP or a doctor. Both clinics offered condoms, oral contraceptives, injectables, implants and IUDs as FP methods, the most used being injectables. All nurses could counsel about FP. Both clinics were supported by a doctor visiting daily.^24,25,26^

### Study population and sampling

All nurses involved with FP as well as female clients attending the two clinics were included. For nurses, inclusion criteria were working at the clinic in any category, i.e., CNP, PN, EN or ENA, and having any experience of FP from counselling to administering contraceptives. An exclusion criterion was working at the clinic for less than 1 year. For clients, inclusion criteria were attending the clinic and having some experience of the FP service (from counselling to having used any method). Exclusion criteria were being pregnant, postpartum (defined as within 42 days post-delivery), and younger than 18 or older than 49 years. Pregnant and postpartum women were excluded because their recent experience with carrying a baby may have a biased influence on their choice to receive FP or not. Criterion-based purposive sampling was used to select information-rich participants and to ensure fair selection.

### Data collection

In total, 18 interviews were conducted (10 with clients and 8 with nurses) from July 2020 to April 2021. The interview guides and processes were piloted using two interviews from each clinic in Afrikaans and Xhosa, respectively, as these are the main languages of people living in the community. These interviews showed rich and appropriate data and were therefore included in the data. Following four nurse interviews, data were updated with emerging themes involving specific methods like condoms and sterilisation, perceived ethnic differences in FP use, opinions on whether young clients should not use FP and why, and personal beliefs about FP. Data saturation occurred after eight client interviews and by the eighth nurse interview.

Data were collected by female research assistants using semi-structured individual interviews in a home language. The reason for research assistants was to overcome any language barriers and power differential that could have existed between the nurse or client and the primary researcher as a male doctor. Furthermore, focus group discussions were not chosen for nurses as power dynamics between levels of nursing staff were anticipated. The average length of the nurse interviews was 42 min while that of the client interviews was 20 min. The research assistants translated and back-translated the interview guides, as well as reviewed them for local language appropriateness.

One research assistant was assigned to each clinic but moved between clinics as needed. Both the research assistants were from the local community, in their late twenties, and were both completing a degree in the medical field at an SA university; therefore, they were familiar with the medical environment and were capable of navigating the complexities of medical field research. One was studying for a degree in community and health psychology. The other was studying for a degree in medical laboratory science. Training of research assistants was done by the primary researcher, including feedback and debriefing after the initial interviews, to help refine the quality and depth of the interviews.

Nurse participants were purposively selected by the primary researcher according to the inclusion criteria and were invited to participate. The interviews were done after-hours at Mossel Bay Hospital’s outpatient department so that no other staff member at the clinic was aware of the nurse’s participation. The nurse was not addressed by name throughout the interview to ensure that the primary researcher also did not know who the participant was. For clients, the research assistant stationed at the clinic recruited participants by directly approaching women at the clinic or receiving referrals from health workers. Selected client participants were interviewed at the clinic in a private room. All participants received refreshments and an honorarium. Corornavirus disease 2019 (COVID-19) precautions were followed. A process for referral to the employee wellness programme or the local social worker was established should it be needed following an interview. An orientation session with the clinic staff was done to ensure support for the research assistant and respect for the confidentiality of participants.

### Data analysis

Audio recordings were translated and transcribed using an external service. Transcripts were anonymised and quality checked by the research assistant who conducted the interview to ensure quality translation and transcription and retention of meaning. Data were backed up and stored securely on a password-protected online drive, only accessible by the primary researcher. Audio recordings were deleted once the study was completed. The transcripts are kept in a secure repository at the University of Stellenbosch should they be requested by readers or journals. The repository is an online, password-protected database, only accessible by the departmental head.

The primary researcher inductively analysed the data with assistance from the supervisor using ATLAS.ti version 8. The framework method was applied (see Table 1).^27^ Trustworthiness of the data was established through various methods (see Table 2).

**TABLE 1:** The framework method.

Number	Steps in the data analysis
1.	Familiarisation occurred through reading all transcripts, reviewing field notes and trying to gain a deep sense of the data.
2.	Codes and categories were formulated, and a thematic index was created. This was reviewed and refined together with the research supervisor.
3.	All transcripts were coded.
4.	The codes were charted in order to gather all the data from the same category in one place.
5.	Each chart was read, and the data were interpreted looking for both the range and strength of themes and their relationships to try and provide an appropriate and coherent explanation of findings. This process was done together with the research supervisor.

*Source:* Pope C, Ziebland S, Mays N. Qualitative research in health care. Analysing qualitative data. BMJ. 2000;320(7227):114–116. https://doi.org/10.1136/bmj.320.7227.114

**TABLE 2:** Trustworthiness of the data.

Element	Description
Credibility	Peer debriefing – researcher debriefed with the supervisor and research assistants debriefed with the researcher. Supervisor with experience in qualitative research supervised the process. Triangulation of data between nurses and clients, as well as between clinics, was done to identify themes that are related to each other, which provides an in-depth understanding.
Transferability	A thick description of the study setting, audit trail and participants is provided. Purposive sampling was done, and data saturation was achieved.
Dependability	Methods employed are documented in detail. Triangulation between nurse and client groups and between clinics was done.
Confirmability and reflexivity	Findings between research assistants at the two respective clinics were triangulated. The primary researcher described his credentials, background and relationship to the objective of the study in a reflexivity statement. Potential bias in the researcher and research assistants was addressed and minimised with reflective discussions between the research assistants and researcher, and the researcher and supervisor.

### Reflexive statement

The primary researcher is a South African male in his thirties working as a Family Medicine registrar in the local sub-district. He has worked at both clinics, as well as in the maternity ward at the district hospital. He is interested in preventing unplanned and unwanted pregnancies by enabling appropriate FP choices and increasing FP uptake at PHC clinics. He believes that social factors and local beliefs highly influence clients’ use of FP as well as nurses’ provisioning of them. In attempting to minimise bias, the researcher frequently reflected on his own judgements, practices, beliefs and assumptions during the study process with the supervisor, which helped him to be reflexive and aware of potential bias.

### Ethical considerations

Ethics approval was granted by the Health Research Ethics Committee of the University of Stellenbosch (S19/07/129). Permission was granted by the Research Committee of the Department of Health of the Western Cape (WC_202003_024), the district manager and the district hospital management. Informed consent was obtained from all study participants.

## Results

Ten clients and eight nurses were individually interviewed. The median age of clients was 28.5 years and of nurses was 47.5 years (see Table 3). Four major themes and some minor themes emerged (see Table 4).

**TABLE 3:** Demographics of study participants.

Participant	Age	Clinic	Relationship status
Client_01	28	D’Almeida	Single
Client_02	27	D’Almeida	In relationship, not living together
Client_03	25	Alma	Married
Client_04	32	D’Almeida	Single
Client_05	21	Alma	Single
Client_06	43	D’Almeida	In relationship, living together
Client_07	33	D’Almeida	Divorced, in relationship, not living together
Client_08	29	Alma	Single
Client_09	19	Alma	In relationship
Client_10	36	Alma	Married
Nurse_01	40s	Alma	-
Nurse_02	30s	Alma	-
Nurse_03	40s	Alma	-
Nurse_04	50s	Alma	-
Nurse_05	50s	D’Almeida	-
Nurse_06	30s	D’Almeida	-
Nurse_07	50s	D’Almeida	-
Nurse_08	40s	D’Almeida	-

**TABLE 4:** Major and minor themes.

Major themes	Minor themes
1. Stigma, culture and the teenage girl	1.1. Moralising and stigmatising FP. 1.2. FP leads to sex, babies lead to FP. 1.3. Culture and FP. 1.4. Teenagers dominate the conversation.
2. Bad effects – the Big Five	2.1. FP makes me fat. 2.2. FP blocks blood or makes me bleed. 2.3. FP causes pain. 2.4. FP messes up my fertility. 2.5. FP might give me cancer.
3. FP social dynamics	3.1. Education and self-determination. 3.2. Caregiver responsibility. 3.3. Rape, abuse and a sense of safety. 3.4. Teen mommies and sugar daddies.
4. FP and the health system	4.1. Clients and nurses value efficiency and respect. 4.2. Dedicated FP resources. 4.3. FP outreach. 4.4. Nurses are kind.

FP, family planning.

### Stigma, culture and the teenage girl

#### Moralising and stigmatising family planning

There was a pervasive stigma about FP that was deeply associated with moral ideas around sex:

‘I think there is still a huge stigma about the prevention [*FP*] story, and that is why I think there are so many teenage pregnancies … the girls are too ashamed to come and fetch the prevention.’ (Client 4, D’Almeida clinic, Age 32)

This stigma was also shared among peers:

‘… there are a lot of young girls whose peers are discussing them [*skinder*] with other friends, [*saying*] that they are now using [*FP*] and [*so*] they are now sexually active …’ (Nurse 4, Alma clinic, Age 50s)

If a teenage client is accessing FP, she was deemed sexually active or even promiscuous:

‘*Hulle is sleg of hulle hou hulle groot* [They are bad or they pretend to be adult], but you need to protect your daughters.’ (Client 4, D’Almeida clinic, Age 32)

‘… when my child comes for the pill or the injections, people are going to talk about it and say that my child is loose before her time.’ (Nurse 8, D’Almeida clinic, Age 40s)

Some were worried that their parents would assume they are sexually active if they use FP:

‘… they are scared because their mommies must not know that they are coming to the clinic because they are not sexually active … if I tell my mother … then [*she*] is going to think that I am sexually active …’ (Nurse 7, D’Almeida clinic, Age 50s)

#### Family planning leads to sex, babies lead to family planning

The majority of clients were first introduced to FP only after their first baby:

‘I was 15 when I fell pregnant … back then parents didn’t talk about prevention. My mother never spoke a word about prevention, but the moment that I gave birth to my child it was only then that I heard anything about prevention.’ (Client 6, D’Almeida clinic Age 43)

There was a belief in the community that FP being used by teenagers led to early sexual debut and gave them ‘permission’ to have sex, or even encouraged it:

‘… they feel ashamed … sometimes parents … that I chat to everyday say that they can’t put their child on FP because it would give their children reason to be sexually active …’ (Nurse 6, D’Almeida clinic, Age 50s)

‘… [*people say*] it does not give you the right to do things that you should not be doing … some people are still old fashioned, they don’t think [*that FP*] is protecting the young girls. Then they complain when the young girls fall pregnant.’ (Client 4, D’Almeida clinic, Age 32)

At the same time, nurses reported that local schools believed that FP leads to sex:

‘… schools don’t actually want us to come and talk about [*FP*]. And I feel that that is actually where it starts … I asked one of the principals … they think that … the children will then start experimenting …’ (Nurse 7, D’Almeida clinic, Age 33)

Some nurses believed that young teenagers should not be engaging in sex and so FP is not needed if abstinence is followed. However, many clients and nurses felt that barriers to teenage FP should not exist, even advocating this from personal experience:

‘But I as a parent feel that … it’s pointless to try and hide it [*FP*] from your child … I know that to be 15 with a child, it’s not a good thing. I am speaking from experience.’ (Client 6, D’Almeida clinic, Age 43)

‘I was a teenage mom and I wish I had that courage to go and say that I want to use FP, but I was too afraid to do that because of the attitudes of nurses … you know, when the client has that first point of contact and she gets that attitude, she is not coming back.’ (Nurse 2, Alma clinic, Age 30s)

A solution lies in what one nurse succinctly said:

‘I really feel that FP should not be hidden. They should do a lot more outreaches … make it fun, don’t present it as a moral, ethical dilemma which puts unnecessary strain on the teenagers.’ (Nurse 8, D’Almeida clinic, Age 40s)

#### Culture and family planning

Half of the nurses reported that differences in cultures influenced those accessing FP. For the purposes of this research, culture was understood to mean an umbrella term encompassing the social behaviour, norms, beliefs, customs and habits found in a society. Differences in requesting FP seemed to come from community-based ideas regarding opportunity, education and self-determination:

‘I think what happens now is that the [*some*] kids, especially women, are being pushed more now towards education so they are of the mindset I need to get educated … like you see them doing engineering and things …’ (Nurse 2, Alma clinic, Age 30s)

‘… it is not a cultural thing, it is just a social thing … there is this mindset in [*some*] communities … you are not going to get into university so why try? You are not going to get that bursary so why apply?’ (Nurse 2, Alma clinic, Age 30s)

Stigma seemed to play a large role in different communities:

‘… there are groups in the community who would bring their daughters to the clinic for FP at an earlier age to prevent them from getting pregnant … I would think it will mostly be [*influenced by*] culture. But like I said there is this stigma around FP …’ (Nurse 8, D’Almeida clinic, Age 40s)

‘When I was growing up my mother never discussed FP with me. I had to learn about that when I started my nursing training. Our people don’t really like to use FP … I suppose it is tradition …’ (Nurse 4, Alma clinic, Age 50s)

Despite the above, some nurses believed that there were no cultural differences regarding accessing FP:

‘All the young ladies are visiting to protect themselves from early or unwanted pregnancies, it is not really race or religion related.’ (Nurse 5, D’Almeida, Age 50s)

#### Teenagers dominate the conversation

Seven of the eight nurses mentioned teenagers at the beginning of their interview, despite the interview guide not mentioning teenagers. The interview guide was set up not to prejudice nurse interviewees towards teenagers but allowing for it to emerge. While this study was limited to adult women of reproductive age and their own experiences, participants were encouraged to express any thoughts that they felt related to FP.

Nurses expressed concern about teenagers under 15 years of age requesting FP. This varied from them believing that such clients are too young to engage in sex, to concerns about the long-term effects of FP on young bodies. Some nurses have turned such clients away without providing FP:

‘Those early ages like twelve, thirteen, I feel like it is still early for them, that’s my opinion. But if they come, you ask her to come with an adult. We are unable to give her at that age.’ (Nurse 1, Alma clinic, Age 40s)

‘… before I administer anything at that age, I always ask why are you on this FP … because you are so young … your womb must grow to become a woman … what are we doing with FP? We’re going now to control this young child’s hormones.’ (Nurse 3, Alma clinic, Age 30s)

Importantly, other clients have noticed this attitude towards young teenagers.

‘… sometimes it is weird when a 14-year-old girl comes in here, the nurses are always worked up about it. But there is a stigma about the whole story.’ (Client 4, D’Almeida clinic, Age 32)

This concern about young teenagers was not universally shared. Other nurses felt that it is good that young people accessed FP, whether they are sexually active or not:

‘… a teenager will come in for [*FP*] for the first time, not sexually active and then some nurses will actually show them away … the fact that she is accessing the service in the first place means she had to be brave … because asking for contraception at a young age is not easy … any teenager who comes to me … [*and wants*] to start … is a bonus for us because we do not have the time to go and find these young girls there outside.’ (Nurse 7, D’Almeida clinic, Age 50s)

### Bad effects – The Big Five

Five minor themes can be clustered around perceived or real side effects of FP (see Table 5).

**TABLE 5:** Perceived or real side effects of family planning.

Minor themes	Quotations
2.1 FP makes me fat: The commonest side effect clients were concerned about was weight gain.	*Then they complained that contraceptives will ruin your body or you will be too fat. You will be having a bad body and your muscles will be loose, so I was afraid of those things. (Client 9)*
	*… a big thing for women when they are using any type of contrac eptive is: am I going to get fat? … I don’t want other people to know … that I am using a type of contraception, so I don’t want to get fat. (Nurse 2)*
2.2 FP blocks blood or makes me bleed: Abnormal bleeding was a common concern among clients.	*… it did not really work for me, do you understand, it did not really help, yes, it only made me feel like I flow more. (Client 7)*
Although some women simply preferred to menstruate, for many amenorrhea was a concern that seemed to emanate from ideas of blood being blocked inside and was also associated with the belief that this was ‘dirty’ or ‘dead’ blood.	*I had a lot of rash all over my face and when I asked my sister … she will say, blood has to come out. It is because of the blood that is not clean … (C lient 9)*
Clients were often concerned about amenorrhea and blocked blood but these were underreported as client concerns by nurses. In contrast, nurses tended to over report abnormal bleeding as a client concern.	*Is it true that because I have back pains, it is because I have blocked the blood that was supposed to go out of my body? It is inside my body because of the injection. (Client 8)*
2.3 FP causes pain: Pain was a common concern, either experienced by self or others, and common symptoms were headaches, back pain and abdominal pain.	*It (Depo-Provera) was right at first and then I had headaches and I felt dizzy. Then I stopped using it. (Client 10)*
2.4 FP messes up my fertility: Clients were concerned about being unable to fall pregnant after using FP.	*… many of the FP methods causes us young people to struggle to fall pregnant … things are not the same … it messes up one’s hormones … when I left it, it took long to fall pregnant. I thought joh (gosh) I can’t have children. But ya, then I bought something from the Rastas (Rastafarians) to clean me. (Client 2)*
	*When she stopped using contraceptives and cleansed herself, till today she didn’t have [more] kids … I am scared if it mig ht happen to me … I must stop using them because I am going to get married … They will say I cannot bear kids of which it is because of the injection. (Client 8)*
Regarding male partners’ influence on FP, they were mainly concerned about fertility, with some worrying about weight gain and blood being blocked.	*He is complaining about the [FP] that it would maybe deprive you not to have a child again … and because he says he wants to have a child you see. (Client 9)*
	*The questions are if she is using it for so long and we want to have a baby, will she be able to have a baby? Is she going to get fat? If her period stays away, does it mean that the blood is pooling inside the body, is there de ad blood inside her – so all the questions that the girls have, the guys also have but they are a lot more [outspoken] (Nurse 2)*
2.5 FP might give me cancer: Clients, as well as some nurses, were concerned about FP causing malignancy.	*They are still young. It has side effects and at a later stage one of these injections makes a person to develop cervical cancer when they are young. (Nurse 1)*
	*My sister was on contraception (injectable) and then they found out that it … made the cancer in her body worse … she used it for a long time … and then [few] months back … they said she has breast cancer … I am very scared … I want another baby and now I am just worried [that] the contraception will destroy everything. (Client 7)*
Two of the eight nurses (one from each clinic) believed that FP in young women led to cancer. One nurse referred to a 50-year-old client who presented with abnormal bleeding and had used FP from her teenage years.	*… and that’s why I’m so concerned when I st art to administer [FP to] young children … you already do harm to the hormones, the uterus, the everything … [sigh] … will this not affect them more to become … to ca cervix (cervical cancer). (Nurse 3)*

FP, family planning.

### Family planning social dynamics

#### Education and self-determination

The use of FP by young women seemed motivated by their desire to study and determine their futures. When asked when was the first time she heard about FP, this client responded with ‘while being at school’ and further said:

‘So that I don’t have a child because I am still young. I had to study first.’ (Client 10, Alma clinic, Age 36)

Nurses reflected on this when asked about the age groups that attend the clinic for FP and why:

‘… like from the 20- year age are women that are becoming more emancipated and liberated so they want to go and study, so they get to choose – they get to plan.’ (Nurse 2, Alma clinic. Age 30s)

‘I think one reason is that they thinking about their future. A lot of our younger community members see how our parents suffered, because they got us so early … We really want to be more successful.’ (Nurse 6, D’Almeida, Age 50s)

When asked whether she thought that FP helped the community, one nurse said:

‘… if you have a teenage daughter in your house and … if she is well-informed and she uses her FP then she can complete her schooling and go for her studies without being kept behind by an unplanned baby or family.’ (Nurse 8, D’Almeida clinic, Age 40s)

#### Caregiver responsibility

Many women reported either their caregivers (mothers, aunts, older sisters) having assisted them with FP, or themselves having assisted their daughters, nieces or younger sisters with FP:

‘My aunt saw that I have grown up. She said I must start using contraceptives.’ (Client 9, Alma clinic, Age 19)

‘I said to my little sisters, come to me if you don’t want to go to your mom about it, then I will go with you [*to the clinic*].’ (Client 4, D’Almeida clinic, Age 32)

Clients and nurses reported that parents are bringing their young daughters to put them on FP:

‘… I am just waiting until she is 14. Then I will put her on it, not that I don’t trust her … you know how life is out there … every parent must do that.’ (Client 6, D’Almeida clinic, Age 43)

‘… parents normally bring their children from the age of 12 years and then they want to let their children use FP to prevent teenage pregnancies.’ (Nurse 4, Alma clinics, Age 50s)

#### Rape, abuse and a sense of safety

Family planning played a big role in providing a sense of safety from the effects of rape in the community:

‘Yes, it (implant) is helping me because anything can happen. Maybe you can be raped, and I know if ever that can happen, I know I am safe …’ (Client 5, Alma clinic, Age 32)

‘… we have a lot of clients, young 12- year- old children that are already on [HIV] medication as a result of sex with older men … maybe they were raped. Rape is quite a big thing in this area. The shebeens are the problem.’ (Nurse 4, Alma clinic, Age 40s)

Family planning provides protection for women in abusive relationships:

‘… when you now sit with the woman … it is like no, my husband threw away my tablets. He wants a baby but I am not ready for a baby … [*then I take her to the sister*] and I will tell her look, this is the situation. This was a chance today to get away …’ (Nurse 2, Alma clinic, Age 30s)

#### Teen mommies and sugar daddies

Nurses reported that some teenagers value fertility and desire pregnancy at a young age:

‘… there was a 15- year- old and she came to check if she was pregnant … she is 15 and the partner is 19 … she actually hoped that she was pregnant … she is in Grade 9 … I actually saw the disappointment in her face … when she found out she is not pregnant … she left without contraceptives.’ (Nurse 7, D’Almeida, Age 50s)

‘There are still a lot of teenage pregnancies and especially in the lower income communities the younger girls think it is an achievement to have a baby …’ (Nurse 5, D’Almeida clinic, Age 50s)

Some nurses reported that teenagers are forced into illegal relationships:

‘… the kids actually do it for the money especially if there is a financially difficult time at home … they go for the older men who might possibly pay them … they come to the clinic as a group who uses the same kind of preventative measures, because the older men pay them for [*it*].’ (Nurse 4, Alma clinic, Age 50s)

### Family planning and the health system

#### Clients and nurses value efficiency and respect

Clients understood the dynamics of a clinic; however, because of work and family demands, they could not accommodate long waiting times. This client asked her 9-year-old to take care of her 5-year-old, lock the doors and wait till she quickly returned from FP:

‘… it really took too long and I also have duties that I need to do at home. I have children waiting for me.’ (Client 1, D’Almeida clinic, Age 28)

Clients felt the long wait to be unnecessary for something quick that they came for frequently. The nurses shared this view:

‘Other times you had to sit and wait which was unnecessary. They could have helped us first. It is just your birth control that you are coming for.’ (Client 2, D’Almeida clinic, Age 27)

‘You find people for FP having to wait for a long time yet FP doesn’t take long. So, it is not really okay. I wish we can do better.’ (Nurse 1, Alma clinic, Age 40s)

Some clients chose to spend money on transport to attend a more efficient clinic or pay for FP by attending a private pharmacy instead:

‘I thought about getting my file transferred [to a nearby clinic], because I don’t have to take a taxi. It is 20, 30 meters from my house … But when I saw that service, I said no way … the service is just not good enough for me.’ (Client 6, D’Almeida clinic, Age 43)

‘I am on chronic medication, so I come every two months to collect my tablets … I work so it will take too long to come to the clinic [for FP] and then go to work. So, I get [FP] there at Clicks (a private pharmacy) and then I walk over the road to work.’ (Client 4, D’Almeida clinic, Age 32)

#### Dedicated family planning resources

When asked how the service can be improved, every nurse from both clinics felt that FP should have dedicated resources, including a specific nurse, room and consulting times:

‘We do not have a specific room where clients can go for FP when they arrive at the clinic, then they have to sit and wait in the queue – FP should be a fast and efficient method of in and out.’ (Nurse 4, Alma clinic, Age 50s)

When asked whether there is enough time to advise clients adequately, the overwhelming answer was no. Nurses valued efficiency and respected their clients’ time:

‘… every point of contact … FP is provided … the client has a one-stop service. If she is coming with her baby that’s sick, the sister in the baby clinic room will administer her FP … if she is coming for a dressing, she can get it there … [*But what about*] the ones just coming in for FP … especially … [*for*] the first time, [*and need*] explaining [*of*] all the options … there is not that time span in the clinic because there is no one specific sister dedicated to do that only.’ (Nurse 2, Alma clinic, Age 30s)

‘In the past … you only produced your clinic card and your name is entered into the register, you don’t have to wait for your file … [*For the*] young girl who comes from school, who has no other diseases or ill health, why can’t we bring back a FP register? [*FP register is simply a book with names and dates of clients receiving FP*.] …’ (Nurse 4, Alma clinic, Age 50s)

#### Family planning outreach

Many nurses suggested that FP outreach should be considered:

‘[*For*] working individuals … assist the clients at their workplace … and also a mobile clinic that can go into the community areas, or in the farming communities … if we could take the [*FP*] facilities to the clients it would really help and make a big difference.’ (Nurse 5, D’Almeida clinic, Age 50s)

#### Nurses are kind

The majority of clients have had a positive experience with nurses regarding FP:

‘She is good and kind … everything she does, she is kind. She has never been rough or maybe shout at me. If I don’t understand English well, she explains till I understand.’ (Client 3, Alma clinic, Age 25)

‘They are always honest, they always tell me what they are going to do, how they will do it. They don’t do things first and then explain to you … I don’t feel uncomfortable asking them something or shy or anything like that … that is what I like about the clinic.’ (Client 6, D’Almeida clinic, Age 43)

Clients felt that nurses took time to answer their questions and even explained reasons for delays:

‘Like the nurses … explained to us now, there are only two of them … there is a sick child … be a little patient, we have a sick patient, we have to just see the older patient first and there was always an explanation …’ (Client 6, D’Almeida clinic, Age 43)

‘When you come on your appointment, they … will take a few minutes to sit down with you and then ask you questions.’ (Client 5, Alma clinic, Age 21)

‘If they give you an injection … if they give you tablets [*and*] you don’t know what it’s for, here they explain everything to you … that is what I like. You don’t have to worry about if the thing will work, or what it is or things like that.’ (Client 6, D’Almeida clinic, Age 43)

## Discussion

The aim of this study was to explore the lived experiences of both nurses and female clients regarding FP services at PHC clinics in a rural sub-district in South Africa. Four major themes emerged: (1) Stigma, culture and the teenage girl; (2) Bad effects – the Big Five, clustered around weight changes, blood blockages and abnormal bleeding, pain, fertility and cancer; (3) FP social dynamics; and (4) FP and the health system.

In a social milieu where teenage sex, and therefore FP, has been highly stigmatised and regarded as immoral, there was a corresponding belief in the community that FP at a young age gave teenagers the impression that sex is appropriate and can be explored. Many nurses, who were from the same community, also held these beliefs and brought them into their practice. Participants also reported that schools prevented FP services on site, while many parents have reported to the nurses that they were concerned their children will be stigmatised if they receive FP. These are not uncommon, as a study in Cape Town about sexual and reproductive health (SRH) described a nurse being conflicted by providing a currently sexually inactive teenager with condoms, as it could be viewed as permission to have sex.^28^ The same study also reported that high schools where the most teenagers requested termination of pregnancy were the ones preventing FP services on their premises.^28^ Furthermore, nurses felt that abstinence was no longer being promoted and should form part of SRH prevention strategies, especially with regard to HIV and sexually transmitted infections.^28^

The majority of clients reported that they first encountered FP after their first child. Paradoxically, nurses believed that FP helped young women to complete their education and determine their futures. Clients in general, and family in particular, supported this idea, many of whom have since assumed responsibility for FP for the younger women in their care. A study on stigma and unmet FP among adolescents in sub-Saharan Africa described internalised stigma, the subjective feeling of being devalued or marginalised, and enacted stigma, the acts that devalue those stigmatised, as culminating in the community idea of the ‘bad girl’.^29^ The community viewed teenagers who participated in sex, as well as those involved in any SRH-related issues like pregnancy, abortion and, importantly, FP, outside marriage as ‘bad girls’.^29^ This stigma extended to her parents, teachers and even religious leaders, with stigma enacted by her peers in the form of gossip and being laughed at being the most damaging.^29^ Therefore, there exists cognitive dissonance in the minds of both parents and nurses regarding the need for FP for teenagers where FP not only is viewed as essential to a bright future but also encourages sex and invites stigma that undermines a bright future. This cognitive dissonance encourages the cycle of believing FP leads to sex, reducing its promotion and uptake, resulting in more unplanned pregnancies, after which FP is then encouraged.

The cultural differences regarding opportunity, education and stigma that emerged seemed to be restricted to the local context as research has not been found elsewhere to corroborate these findings. However, a 2004 study looking at contraceptive use in late-and post-apartheid South Africa found that among some groups of sexually active women who have never been married, the use of FP before marriage was high, while for other groups of women, this was found to be low. After marriage, for both groups, FP use rose after the birth of the first child.^30^

The majority of bad effects from FP centred around the five minor themes. As reported in other research, bad effects included both true side effects and myths, and clients learnt about these from family and friends, rather than nurses.^13,31^ Similarly, concerns around weight changes dominated, including losing weight.^31^ Abnormal bleeding mainly included menorrhagia and withdrawal bleeds. Amenorrhea was not universally reported as a bad effect, and many women used FP to induce amenorrhea as a preference, including those not sexually active. ‘Pain’ centred around headaches, back and lower abdominal pain, and many clients associated them with a belief that FP was causing some form of blood blocking, hormonal or anatomical harm. The idea of blood blocking is frequently reported in research.^15,31^

Family planning impairing fertility was a common concern among both younger and older women and resulted from ideas of hormonal or anatomical harm from FP. Some nurses also shared this concern. A study exploring sexual risk behaviour perspectives of teenage mothers in rural KwaZulu-Natal also found that many believed FP caused sterility.^32^ Concerns around cervical cancer were held by some clients and some nurses. This was attributed to the injectable contraceptive in some cases which was also found in other research.^31^ Clients tended not to distinguish bad effects between various FP methods. Some knew about Depo Provera^©^ causing weight gain, for example. However, most clients had a baseline concern about FP that increased from the most popular (injectables) to the least popular methods (LARCs).

This study showed that parents were so concerned about teenage pregnancies that they brought their children for FP irrespective of whether the teenager was sexually active. The nurses, also concerned about teenage pregnancies, were inhibited by concerns about biological harm and perhaps a latent belief that teenagers should not be engaging in sex. These nurse beliefs represent barriers to FP uptake with abstinence being advised instead of FP, resulting in teenagers being left relatively unprotected from pregnancy.^28,29,31,32^

Family planning was regarded as protective from the effects of rape, intimate partner abuse and illegal teenage relationships with older men. Fertility was highly valued at a young age and also among the most vulnerable women, especially those in so-called transactional relationships, where a pregnancy or child was used to secure her future with a partner. Family planning should play a greater role in such social dynamics, but received more stigma as, in the hope of protecting young women, people advocated ideas like abstaining from premarital sex, thus moralising sex and FP. Male partners influence FP, and as found in this study, clients may have to use FP without disclosing to their partner. This was also found in a study focused on male partner influences on FP.^33^ A recommendation from that study was that involving the male partner in FP during the consultation could be valuable in improving access and adherence to FP.^33^ Healthcare workers should accept the individual and contextual realities of clients requesting FP and should have a family-oriented approach, and consider involving the partners, with voluntary client consent. The finding of teenagers engaging in transactional sex with older men (referred to as ‘blessers’) has also been described, and this phenomenon, although clearly illegal, seemed to be normalised among teenagers.^32^ Healthcare workers have an obligation to report such activities and advocate for vulnerable women.

Nurses unanimously suggested that dedicated FP resources could improve the FP service at their clinic. This has been echoed in research where separate space and timeslots were needed to cater for the SRH needs of teenagers.^28^ Clients believed FP was uncomplicated, quick and a frequent healthcare need, and consequently deemed long waiting times inappropriate, given work and family commitments. Nurses agreed with this and understood that although some clients had a complicated need or were unwell, the majority needed help quickly and efficiently. Enabling FP at every point of contact improved access for some but did not improve access for the client coming solely for FP, especially the first-time FP client. Clients also sympathised with nurses who were working hard but were unappreciated or even ridiculed by some clients who were frustrated by long waiting times. Time pressure was the most serious challenge facing nurses providing SRH services, where the healthcare system was perceived as favouring quantity over quality, with limited regard for complex consultations.^28^ Clients in this study regarded the nurses as kind, approachable and caring, which was in contrast to other studies.^14,15,29^ However, this may be because of the exclusion of clients below the age of 18.

Family planning is viewed in a disease-focussed mindset and finds itself in a health system that is ‘sick role-centric’, focused on treating disease rather than promoting and maintaining health.^34^ Comprehensive PHC results in FP being strongly coupled to disease prevention, including HIV testing, breast examination and cervical cancer screening.^7,35^ Clients have to wait in queues along with those attending the clinic for disease screening, disease management or ill-health. Consequent time pressures result in quick explanation of side effects from a biological perspective rather than taking time to address social beliefs that are often complex and remain deeply entrenched and prevalent. Subsequently, informed consent, and therefore client autonomy, is compromised. In order to improve FP uptake, the social milieu of individuals and SRH stigma need to be considered.^29^

At the Global Conference on PHC, the Declaration of Astana envisioned PHC and health services that are provided with compassion, respect and dignity, as well as enabling empowering and healthy environments that maintain health and well-being.^36^ As seen in this study, the experiences of FP shed light on how SRH forms part of the social foundation of society.^37^ If FP is a key to self-determination, then penalising the young, the healthy and the employed who use FP regularly is not aligned to this vision. Empowering individuals and communities to maintain their health through a socially responsive health system is aligned to this vision.^38^

### Limitations

COVID-19 delayed research approval, data collection and analysis. Data collection for nurses was deferred to after a COVID-19 wave because of staff shortages and staff burnout which prevented concurrent data collection and analysis as initially planned. Given this delay, member checking, respondent validation and negative case analysis were not done which limit the credibility of the data analysis. It was difficult to find older clients who were willing to participate, with only one out of the eight clients interviewed being above 36 years of age. No nurses between the ages of 18 to 29 years agreed to participate despite multiple attempts at setting up interviews with them. This may have provided unique information on the FP experiences of older clients and younger nurses, respectively. Because of scope constraints on the project, women below the age of 18 years were excluded. However, the data showed that nurses in particular were concerned about teenagers when it came to FP, and clients viewed experiences with nurses as positive, which may have not been the case for younger teenagers. This should be explored in future research.

### Recommendations

A guideline should be formulated to enable nurses to effectively and appropriately manage teenagers or their parents requesting FP. No client requesting FP should be turned away.

The Big Five bad effects, and not just biological side effects, need to be focused on and actively addressed in FP education and counselling, including appropriate training for all nurses before they provide any form of FP service, even just counselling.

Dedicated FP resources (a combination, in part or all, of nurse, room and time slots) should be encouraged at clinics, especially for clients presenting specifically for FP. Family planning outreach into communities should be provided.

Topics for future research include exploring teenage clients’ and nurses’ experiences regarding FP; exploring the beliefs, ideas and attitudes that teachers and parents of school-going children have about FP; and using community-oriented primary care principles to address FP stigma in the community.

## Conclusion

This study explored the lived experiences of nurses and female clients regarding FP services at PHC clinics in a rural sub-district in South Africa. Family planning is highly moralised and stigmatised with a prevailing idea that FP leads to sex among teenagers. In contrast, FP is also used by sexually inactive women and is viewed as beneficial in completing education and enabling self-determination. Family planning outreach into the community and dedicated FP resources at clinics were suggestions to improve the service. These findings may be useful to inform FP services in similar PHC contexts in sub-Saharan Africa and elsewhere.
